# Interactions of boron nitride nanosheet with amino acids of differential polarity

**DOI:** 10.1038/s41598-022-13738-5

**Published:** 2022-07-01

**Authors:** Fatemeh Najafi, Farzaneh Farzad, Samaneh Pasban

**Affiliations:** Department of Chemistry, University of Birjand, Birjand, Iran

**Keywords:** Biochemistry, Biological techniques, Computational biology and bioinformatics

## Abstract

Free amino acids represent a category of different biomolecules in the blood plasma, which bond together to make up larger organic molecules such as peptides and proteins. Their interactions with biocompatible nanoparticles are especially important for plasma-related biomedical applications. Among the various nanomaterials, the applications of carbon and boron nitride-based nanotubes/nanosheets have shown a huge increase in recent years. The effect of molecular polarity on the interaction between a boron nitride nanosheet (BNNS) and amino acids is investigated with quantum mechanical calculations by density functional theory (DFT), classical MD simulations, and well-tempered metadynamics simulations. Four representative amino acids, namely, alanine (Ala), a nonpolar amino acid, and aspartic acid (Asp), lysine (Lys) and serine (Ser), three polar amino acids are considered for their interactions with BNNS. In DFT calculations, the values of the adsorption energies for Lys-BNNS and Ser-BNNS complexes are − 48.32 and − 32.89 kJ/mol, respectively, which are more stable than the other cases. Besides, the adsorption energy calculated confirms the exergonic reactions for all investigated systems; it implied that the interaction is favorable electronically. The MD results show that the LYS molecules have a higher attraction toward BNNS because of its alkane tail in its side chain, and the ASP revealed the repulsion force originating from its COO– group. All the results are confirmed by free energy analyzes in which the LYS showed the highest adsorption free energy at a relatively farther distance than other complexes. In fact, our results revealed the contribution of functional groups and backbone of the amino acids in the adsorption or repulsion features of the studied systems.

## Introduction

Recent advances in biochemistry lead to the production of new biomaterials that can be used for multiple purposes^1,2^. For example, biologically developed materials in particular systems have more capabilities and better performances. Some of such newly developed materials can accelerate the healing process or be used as a biocompatible replacement for damaged tissues or organs. Assuredly, their entry into the body may cause some defects, and accordingly, biocompatibility must be achieved after insertion into the physiological environments so that their advantages outweigh their disadvantages. Overall, the production of biological substances is improving our lifestyle, enhancing our healthy living standards, and consequentially raised our life expectancy^3,4^. To keep up with new challenges and to gain the most from the current opportunities, scientists are supposed to propose new biocompatible nanomaterials^5–7^. Since molecular dynamics (MD) simulation is a science that studies the evolution of chemical systems at the nanoscale, it can provide us with a nanoscopic frame of view into the biochemical reactions^8–10^. On the other hand, nanotechnology deals with the production and usage of materials at the nanoscale, therefore, its convergence with MD simulations will considerably contribute to the advancement of nano-biomedicine^11–14^.

Two-dimensional (2D) nanomaterials such as graphene have been extensively explored to understand their capabilities in biological environments^15,16^. Due to its large area and excellent electronic conductivity, graphene has found its way into the drug delivery systems too^17^. Limited solubility and some toxicity side effects forced scientists to search for alternatives to this highly capable nanomaterial. The boron nitride nanosheets (BNNSs) consist of B and N atoms that are attached via covalent bonds in sp2 hybridization^18^. The resulted configuration is a honeycomb structure, which is very similar to that of graphene nanosheet. Because of its biocompatibility, nontoxicity, chemical inertness, and higher solubility in comparison with carbon-based nanomaterial, BNNSs are increasingly received more and more attention in biomedical researches and industries^19^. Since, the superior advantages of boron nitride nanosheets over graphene are its increased water solubility, and non-toxicity, therefore it can make a better nano-vehicle to carry drugs and genes to the needy organs, tissues, or cells in the human body^20,21^. Gnatyuk et al., used 2D boron nitride nanoparticles for the carrier system of the anticancer drug doxorubicin (DOX)^22^. Permyakova and colleagues used high-potential boron nitride nanocarriers for drug delivery^23^. Garcia-Toral et al.’s modifications on the surface of nanoparticles via various ligands have shown to be a successful strategy to turn the substrates into a specific delivery agent to treat tumor diseases^6^.

Amino acids are among the most important constituents of a living body^24^. The most characteristic features in amino acids are functional groups, including amine and carboxyl, attached to the side chain of various types^25^. While a single amino acid can move freely and swiftly in biological environments^26^, it can be easily absorbed by living substances or cellular receivers to deliver a message. They can also form peptides and proteins as they are attached. Proteins play essential roles in the biological system, such as enzymatic, signaling, and transport units in cells^27,28^. Such characteristic features revealed by amino acids suggest that if one can insert them artificially into the cells, it can control the behaviors of the living cells. This is the basics of tissue engineering, in which amino acids are loaded on some biocompatible carrier to deliver into the specific cells to initiate the desired responses. Accordingly, explorations on the adsorption/desorption of proteins on/from synthetic biomaterials are of great importance. Nowadays, computational works have their focus on the amino acid side groups, because the main chain is where the whole body of amino acids is carried out. Sultan et al.^29^ performed atomistic MD simulation alongside metadynamics to derive free energy of adsorption for different amino acid side chains at the negatively charged titania. Their results predicted that in the neutral pH, charge amino acids shows higher affinities toward titania surface, while interaction between substrate and the uncharged amino acids found to be weak or even repulsive. In a particular case, Yazdan yar et al.^26^ investigated the adsorption of functional groups (polar, nonpolar, positively charged, and negatively charged) on the surface of rutile crystal. Their free energy calculations through the metadynamics simulations revealed that irrespective of the side groups, the backbone can be easily absorbed on the negatively charged rutile surface, but for the side chains, group’s adsorption can only be seen for polar and charged.

**Figure 1:**
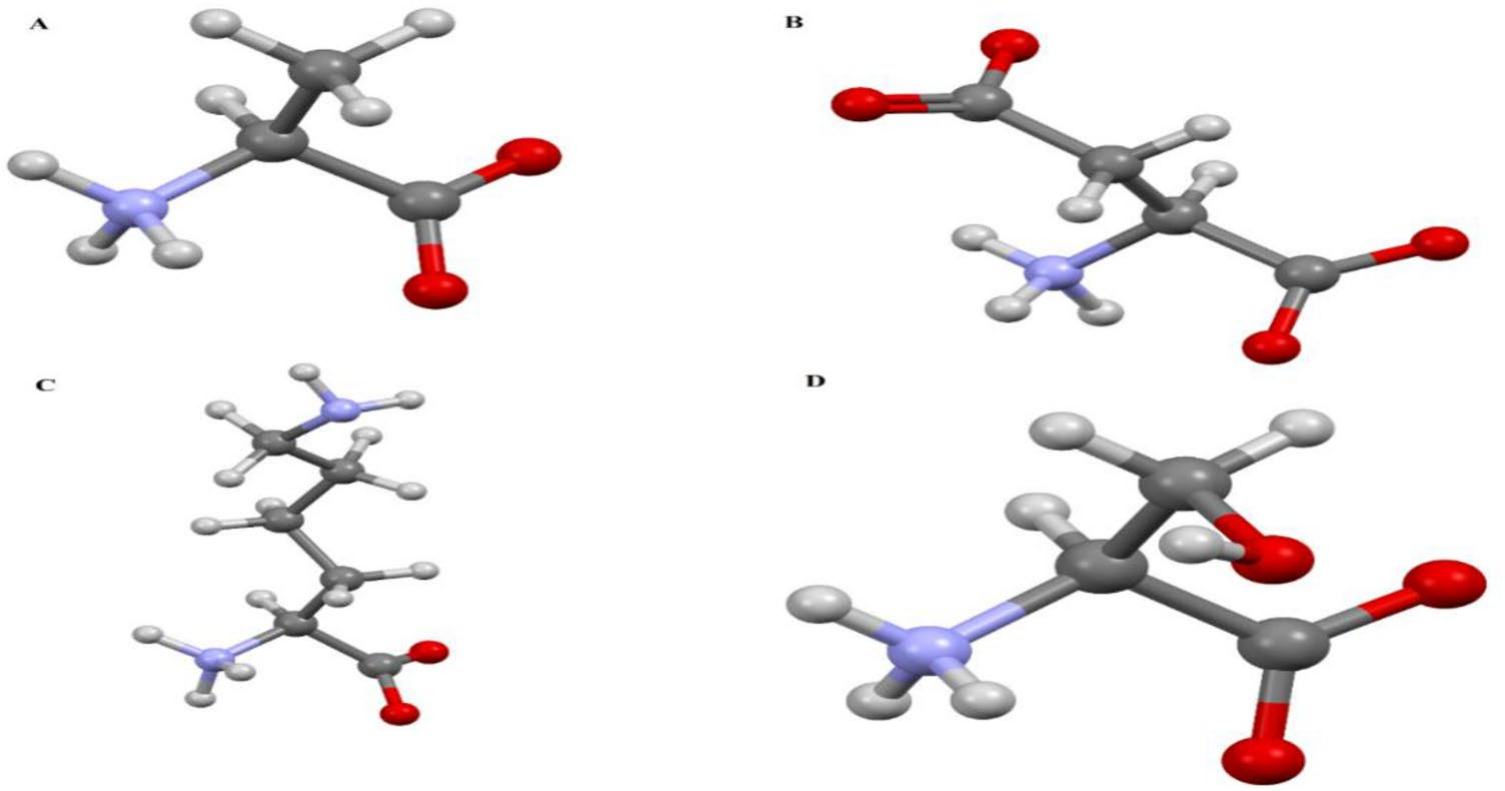
Ball and stick representative of the amino acids investigated in this paper. (**A**) *ALA*, (**B**) ASP, (**C**) LYS, and (**D**) SER [atomic symbols: N (blue), O (red), C (gray), and H (white)].

In this work, the DFT calculations are carried out to obtain the adsorption energies and geometries of amino acid/BNNS systems in the solvent phase. Furthermore, the MD and metadynamics standpoint are used to gain deep insight from the dynamic and diffusion properties of four amino acids adsorption on the surface of boron nitride with a slight negative charged carrier in a biological environment. The selection of amino acids is made in a way to consider amino acids with different side groups (Table 1; Fig. 1). Considering the full amino acid, we would like to find out whether it is the side groups or the backbone, which mainly govern the adsorption. The chosen amino acids have non-polar (ALA) and polar (SER) functional groups with positive (LYS) and negative (ASP) charges in their side chains. Accordingly, this paper hoped to shed light on the differences that are made by a variety of side change and/or backbone on the interactions of amino acids with BNNS for tissue engineering purposes.

**Table 1:** A summary of the characteristics of the amino acids explored in this investigation.

Amino acid	Code		Side chain			Molecular mass	Abundance in proteins (%)
	1	3	Class	Polarity	Charge		
Alanine	A	Ala	Aliphatic	Nonpolar	Neutral	89.094	8.76
Aspartic acid	D	Asp	Acid	Acidic polar	Negative	133.04	5.49
Lysine	K	Lys	Basic	Basic polar	Positive	146.189	5.19
Serine	S	Ser	Hydroxylic	Polar	Neutral	105.093	7.14

## Computational methods

### Quantum mechanics calculations

The computational model of BNNS consists of 48 boron and 48 nitrogen atoms in a similar hexagonal pattern with the dimensions of 15 Å × 17 Å. This nanosheet is saturated with the desirable number of hydrogen atoms to eliminate the dangling bonds. Lys, Ser, Ala, and the Asp amino acids are modeled in their physiological conditions.

In the present work, the selected structures of the host/guest inclusion complexes are optimized using the DFT calculations and the hybrid meta exchange–correlation M06-2X functional^30^ along with the 6-31G** basis set^31,32^. While the M06-2X hybrid meta-functional precisely predicts accurate valence and weak interactions via including dispersion correction. It is worth mentioning the conformational search, which is carried out by the Spartan program package, is used to find the best configuration of amino acid-carrier complexes. The default self-consistent reaction field (SCRF) method^33^, according to the polarizable continuum model (PCM)^34^, is applied to take into account the solvent effect of water. All theoretical calculations are done using the GAUSSIAN 09 computational package^35^. Adsorption strengths are determined by computing the adsorption energy (Eads) that can be obtained from the following equation:

1 $$E_{ads} = E_{BNNS + amino\,acid} - \left( {E_{BNNS} + E_{amino\,acid} } \right)$$

where E_BNNS+amino acid_, E_BNNS_, and E_amino acid_ are the energy of host–guest complexes, isolated BNNS and isolated amino acid, respectively.

Moreover, the stability of host/guest complexes has been evaluated via calculating the solvation energy by using the Eq. (2).

2 $$\Delta E_{solvation} = E_{sol} - E_{gas}$$

where ∆E_solvation_ is the solvation energy of the system and is comprised of two energy terms: where E_sol_ is the total energy of the system in the solvent phase, and E_gas_ is the total energy of the system in the gas phase.

### Molecular dynamics simulations

Four simulation boxes with dimensions of 6 nm × 6 nm × 5 nm are designed in which every box contains a BNNS as substrate and ten amino acid molecules of one type as adsorbents (types of amino acids; alanine(Ala), serine(Ser), aspartic acid(Asp), and lysine(Lys)). BNNS with dimension of 4 × 4 nm^2^ is placed in the center of the simulation box, and then the amino acids situated at least 2 nm away from the BNNS surface. All systems are filled with TIP3P water model molecules. The Na^+^ and Cl^−^ ions with the concentration of 0.15 M are added to neutralize systems and provide a similar biological medium. A detailed understanding of the force field parameters and atomic charges associated with the boron (B) and nitrogen (N) atoms focuses on two key parameters: sigma (σ), which indicates the distance where potential energy is zero and affects atomic proximity, and epsilon (ϵ), which measures the strength of attraction between atoms. Both parameters are essential for accurately simulating interactions in boron nitride. These parameters were extracted from studies conducted by Wu et al.^36^. Also, the parameters for amino acids utilized were obtained through the SwissParam web server (available https://www.swissparam.ch/). All MD simulations are performed by using GROMACS version 2019.2^37,38^. Temperature and pressure are maintained at the biological condition of 310 K and 1 atm via a v-rescale thermostat^39^ and Parrinello-Rahman barostat^40^ algorithms, respectively. The Newton equation of motion of the atoms is measured using the leap-frog algorithm in every time step. The simulations are carried out with a time step of 1.5 fs for 105 ns. During the equilibration, all bonds are kept frozen via the LINCS algorithm, and also the Grid algorithm is used to search for neighboring atoms. The cut-off for the short-range van der Waals interaction is specified at 1.2 nm and the long-range electrostatic interactions are represented via the particle-mesh Ewals (PME) method^41^. It is noteworthy that no external force has been used to adsorb of all the amino acids on the boron nitride sheet, and the charge of the studied systems are zero. Molecular visualizations are performed via visual molecular dynamic (VMD)^42^.

### Metadynamics simulations method

The free energy calculations for adsorption of amino acid analogs on the BNNS are carried out via well-tempered metadynamics simulation by the sum-hills tools in PLUMED plugin 40 v. 2.5.2^43^ patched to Gromacs 2019.2. To do so, four simulation boxes with dimensions of 4.4 nm × 4.4 nm × 7 nm are designed, in which there are a BNNS and the amino acid of interest. The distances between the centers of mass (COMs) from the amino acid analogs to BNNS are taken as the reaction coordinates (CVs) in all the modeled systems. A well-tempered metadynamics bias factor of 15 is used in all simulations with an initial height of 1.0 kJ/mol of Gaussian hills with the width 0.5 for both CVs, which were deposited every 500 time-steps.

## Results and discussion

### DFT calculation results

A complete understanding of the nature of the interaction between the BNNS and amino acid should contain the consideration of all 20 amino acid molecules interacting with BNNS. However, achieving the optimized configurations of all amino acids would have been prohibitively computationally expensive. Instead, four representative molecules belonging to all four prevalent classes are considered, as mentioned above (Fig. 1). In this section, the interaction between Lys, Ser, Ala, and Asp amino acids and BNNS nanosheet are examined, and the equilibrium configurations of the conjugated complexes are analyzed in terms of binding energy, solvation energy, and bond distances. Figure S1, supplementary information, presents the equilibrium configurations of Asp-BNNS, Lys-BNNS, Ser-BNNS, and Ala-BNNS complexes. To assess the suitability of the BNNS towards the adsorption of amino acids, adsorption energy (∆E_ads_) is a key parameter that has been computed using Eq. (1). The obtained adsorption energy values and the important distances of each complex from DFT calculations are given in Table 2. As can be seen In this table, the shortest distances belong to the optimized Lys and Ser complexes, where the hydrogen bonds are formed between the surface nitrogen’s and hydrogens of the –NH, –OH groups, and the calculated distances between these atoms are 2.46 and 2.31 Å, respectively. In Table 2, the negative adsorption energies for all complexes indicate that the adsorption process is exothermic in the solvent phase. These values propose that Lys, Ser, Ala, and Asp amino acids are stabilized by the BNNS surface and the adsorption of these amino acids experimentally possible on the BNNS surface from the energetic viewpoint. The interaction strength of Lys, Ser, Asp, and Ala amino acids on the BNNS surface depends on the chemical nature of amino acids; the basic Lys, polar Ser, and acidic Asp have higher binding energies than nonpolar Ala. However, it found that the adsorption energy is maximum for larger-sized LYS amino acid, which indicates the increase of thermodynamic stability of this system. Table 2 reveal that the values of solvation energies are negative for all investigated systems indicating that solvation of complexes is spontaneous and implicate that water stabilizes the BNNS/amino acids complex. It should be pointed out during adsorption; the E_ads_ values of amino acids have different ranges, indicating that the adsorption capability of different amino acids has significant differences. It is worth noting that a similar hierarchy in the order of the binding energy is obtained for a CNT^44^, though the range is much smaller than that of a conjugated BNNS complex. As a result, semiconducting BNNS are expected to be more sensitive to amino acid molecules and have a higher distinction ability than CNTs.

**Table 2:** Amino acid-conjugated BNNS in the solvated phase: adsorption energy Eads, solvation energy ΔE_solvation_ (all in kJ/mol), nearest-neighbor distance of different complexes obtained using the DFT (M06-2X/6-31G**) level of theory.

Complex	E_ads_	ΔE_solvation_	Nearest-neighbor distance	R (Å)
Lys-BNNS	− 48.32	− 182.10	R(NH(Ala)–N(BNNS)	2.46
			R(NH(Ala)–N(BNNS)	2.63
			R(CH(Ala)–N(BNNS)	2.72
Ser-BNNS	− 32.89	− 153.32	R(NH(Ala)–N(BNNS)	2.77
			R(OH(Ala)–N(BNNS)	2.31
			R(CH(Ala)–N(BNNS)	2.71
Asp-BNNS	− 18.13	− 99.51	R(NH(Ala)–N(BNNS)	2.64
			R(CH(Ala)–N(BNNS)	2.71
Ala-BNNS	− 17.04	− 92.01	R(NH(Ala)–N(BNNS)	2.5
			R(CH(Ala)–N(BNNS)	2.77

### MD simulations

**Figure 2:**
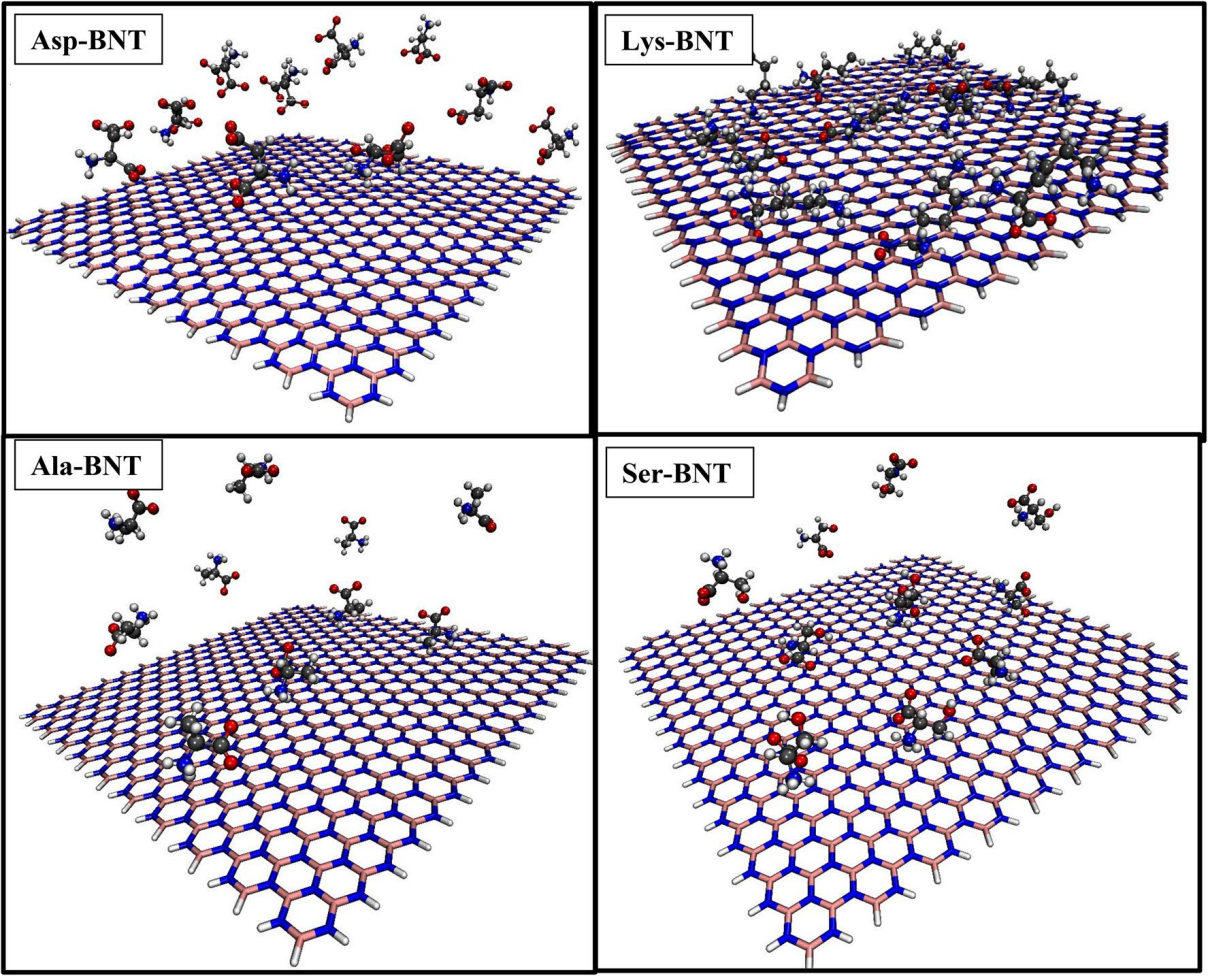
Final snapshots of investigated systems obtained from MD simulations [atomic symbols: N (blue), O (red), C (gray), B (pink), and H (white)].

Considering the different natures of the amino acids in aqueous environments, their interactions with absorbents may vary from one substrate to another. For the case of the BNNS, which is the substrate of interest in this work, the final snapshots of the studied systems are presented in Fig. 2.

**Figure 3:**
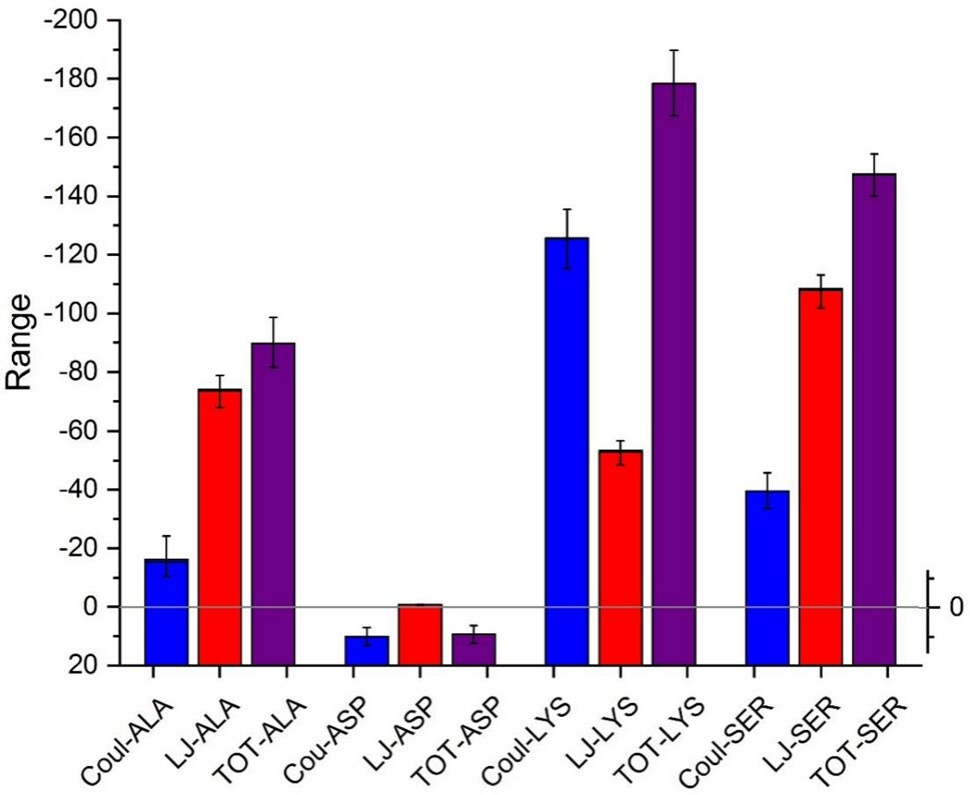
The average coulombic, van der Waals, and total interaction energies between different components of the studied systems and the error bars represent the standard deviations of the data.

Close inspection of this figure shows that LYS, ALA, SER amino acids have more tendency to interact with the BNNS compared to that of ASP. This feature might be related to the ASP’s negative charge in its side chain. This preconception will be evaluated through many informative analyses as follow. The initial and final interactions energies between studied amino acids and the substrate are averaged and provided in Table 3. The averaged interaction energies for the last five ns of each simulation process are also projected in Fig. 3. This figure reveals that interactions between BNNS and ASP are repulsive, and accordingly, it could not be adsorbed on the Boron nitride nanosheet. In fact, there are no L–J interactions between BNNS and ASP, while the repulsive force is in the forms of coulombic (Fig. 3). Accordingly, it can be concluded that the repulsive electrostatics interactions between the charge point on the carrier surface and the side group of Asp, made its adsorption less favorable. On the other hand, the interactions between BNNS with ALA, SER, and LYS are attractive in a way that LYS shows the strongest interaction energies. For ALA and SER, the highest interaction is the L–J term. For ALA and SER, the highest interaction is related to the L–J term, while for LYS, which has the most adsorption energy, the coulombic term plays a crucial role. Interestingly, many other experimental and computational studies reported the same results and confirmed that the nature of such interactions are essentially coulombic. As an example, Roddick-Lanzilotta et al.^45^ explored that in the interaction of positively charged LYS with a negatively charged titanium oxide surface, the electrostatic interaction dominants in the neutral pH. Tentorio et al.^46^, also showed that the amorphous titanium oxide nanoparticles can adsorb GLU and LYS solely through coulombic interactions.

**Table 3:** The L–J, electrostatic and total energies (all in (kJ/mol)) of four study systems and SD represent the standard deviations of the data.

System	Electrostatic			L–J			Total	
	0–5 (ns)	100–105 (ns)	SD 100–105 (ns)	0–5 (ns)	100–105 (ns)	SD 100–105 (ns)	0–5 (ns)	100–105 (ns)
BNNS/Ala	− 11.40	− 16.24	5.107	− 70.96	− 73.59	4.32	− 82.37	− 89.84
BNNS/Asp	3.073	10.13	2.42	− 33.29	− 0.75	0.07	− 30.22	9.38
BNNS/Lys	− 139.76	− 125.56	7.74	− 57.56	− 52.90	3.33	− 197.32	− 178.476
BNNS/Ser	0.37	− 39.524	4.81	− 104.5	− 107.78	4.56	− 104.16	− 147.30

**Figure 4:**
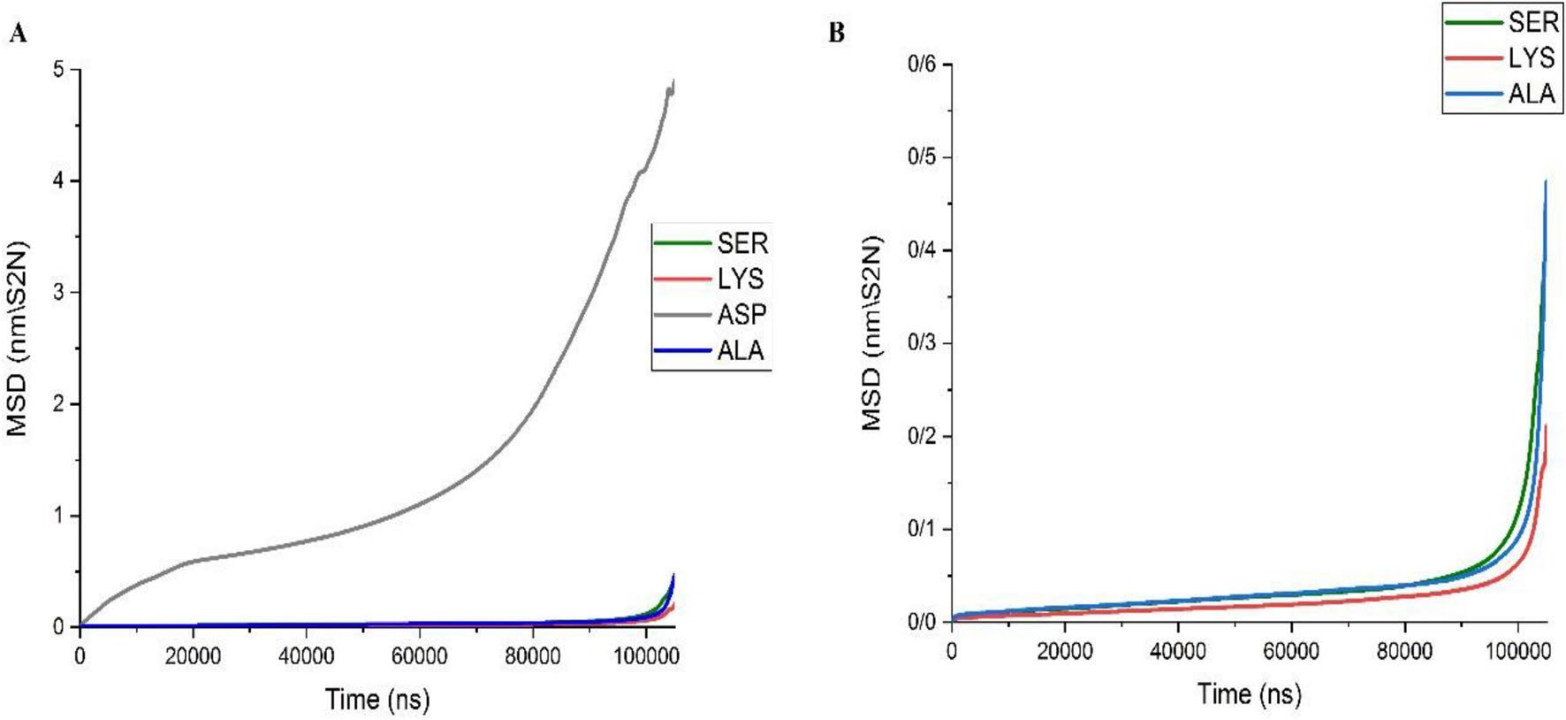
The MSD curves for the amino acids investigated in the simulated systems.

These results can be better presented via calculations of the Mean Squared Displacements (MSD). The MSD values are steadily increasing through the passage of the simulation time for a freely moving molecular entity. However, the extent of displacement of an adsorbed molecule is somehow restricted to its vibrational movements. Therefore, its MSD curve will not show variation through the simulation time. Figure 4 presents the MSD curves for the amino acids investigated in the simulated systems. As can be suggested from the interaction energy values, the MSD curve for ASP locates at a much higher position compared to other amino acids. This phenomenon is the result of the repulsive interactions between the negatively charged protein and the partially negative charged substrate. In fact, it means that the strongly adsorbed water molecules on the surface of both entities shield the interactions between the carrier, and charged functional groups of the amino acid. These features can lead to the free movements of the ASP within the solvent medium (high MSD values). However, the adsorbed SER, LYS, and ALA have similar curves and show an abrupt decrease in comparison to the MSD curve of ASP (Fig. 4A, B). Interesting, for Lys, that its side group is slightly longer, its flexibility compared to two others amino acids is slightly reduced.

**Figure 5:**
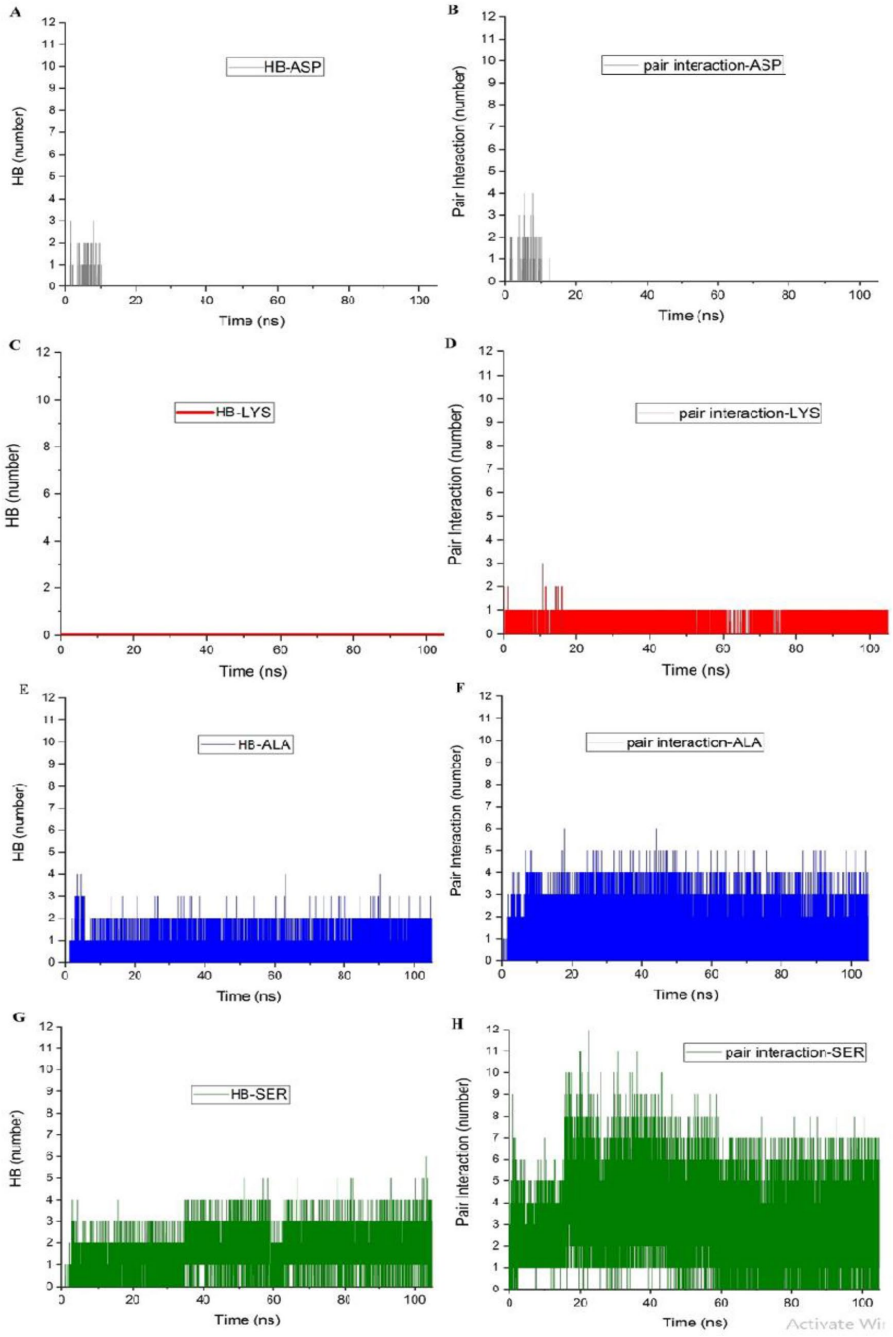
The number of HB and Pair interactions between the amino acids and the BNNS nanosheet.

Furthermore, the extent of coulombic interactions are usually reflected in the number of possible Hydrogen Bond (HB) formed between the amino acids and the substrate. Therefore, the number of HB and Pair interactions between the amino acids of interest and the BNNS nanosheet are calculated and presented in Fig. 5. Close inspection of this figure indicates that for the ASP amino acid, which shows the coulombic interaction equal to zero at the last five ns of the simulations, the number of HB and pair interactions are also equal to zero for much of the simulation time. However, there is a limited number of HBs at the beginning of the simulation, which might be the result of the random movement of the ASP molecules in the simulation box. For ALA and SER molecules that show relatively higher values of coulombic interactions, the number of HB and Pair interactions are relatively more. In fact, in Lys/BNNS system, the formation of the hydrogen bond may be further attributed to the interaction between the hydroxyl group on the side chains of serine and nitrogen atoms on the BNNS surface. In the case of the Ala/BNNS system, it is probable that hydrogen bonds formed between the NH3 group of the backbone of the amino acid alanine and nitrogen atom on the BNNS surface. However, it should be mentioned that the number of HB and Pair interactions are not in full compliance with the coulombic interaction for LYS molecules. This finding means that there could also be other factors that control the extents of coulombic interactions in the studied systems. The reason for the limited number of pair interactions found in systems with the highest values of coulombic interaction might be the fact that the calculation of pair interactions requires a cut-off distance equal to 0.35 nm. This preconception means that interactions between positively charged amino acid and surfaces carrier at the equilibrium states might be more than 0.35, and consequently will not be measured.

**Figure 6:**
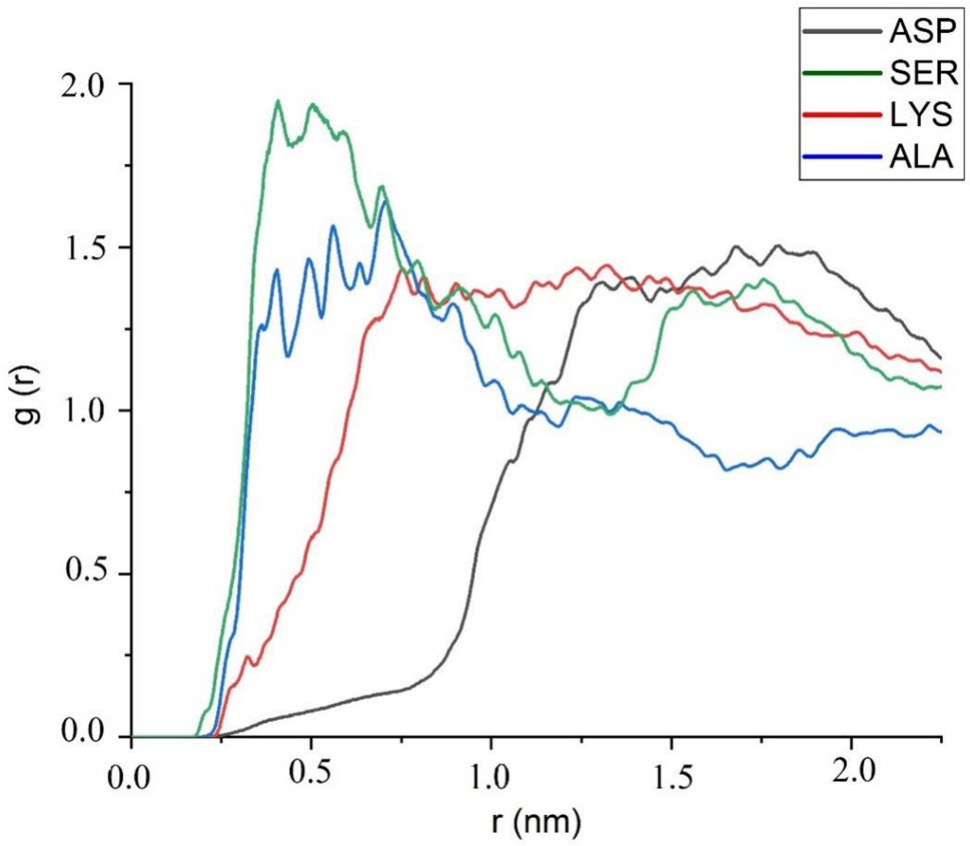
Radial distribution function of the amino acids around the BNNS surface in the study systems.

The distances between the amino acids and BNNS can be examined through analysis of the distribution of the molecules around the substrate. In this regard, Radial Distribution Function (RDF) can provide us with an understanding of the spatial accumulation of amino acids concerning the center of mass of the BNNS. Results of RDF analysis in the studied systems are provided in Fig. 6. In this figure, it can be seen that the highest accumulations of SER and ALA, which formed higher numbers of HB and pair interactions with the substrate, are located at 0.50 to 0.75 nm away from the BNNS surface. While for ASP and LYS molecules, which show lower numbers of HB and pair interactions, the equilibrium states are found at the longer distances away from the BNNS surface. This finding confirmed that there are more chances for SER and ALA molecules, which could be located close to the BNNS surface, to form HB and pair interactions.

**Figure 7:**
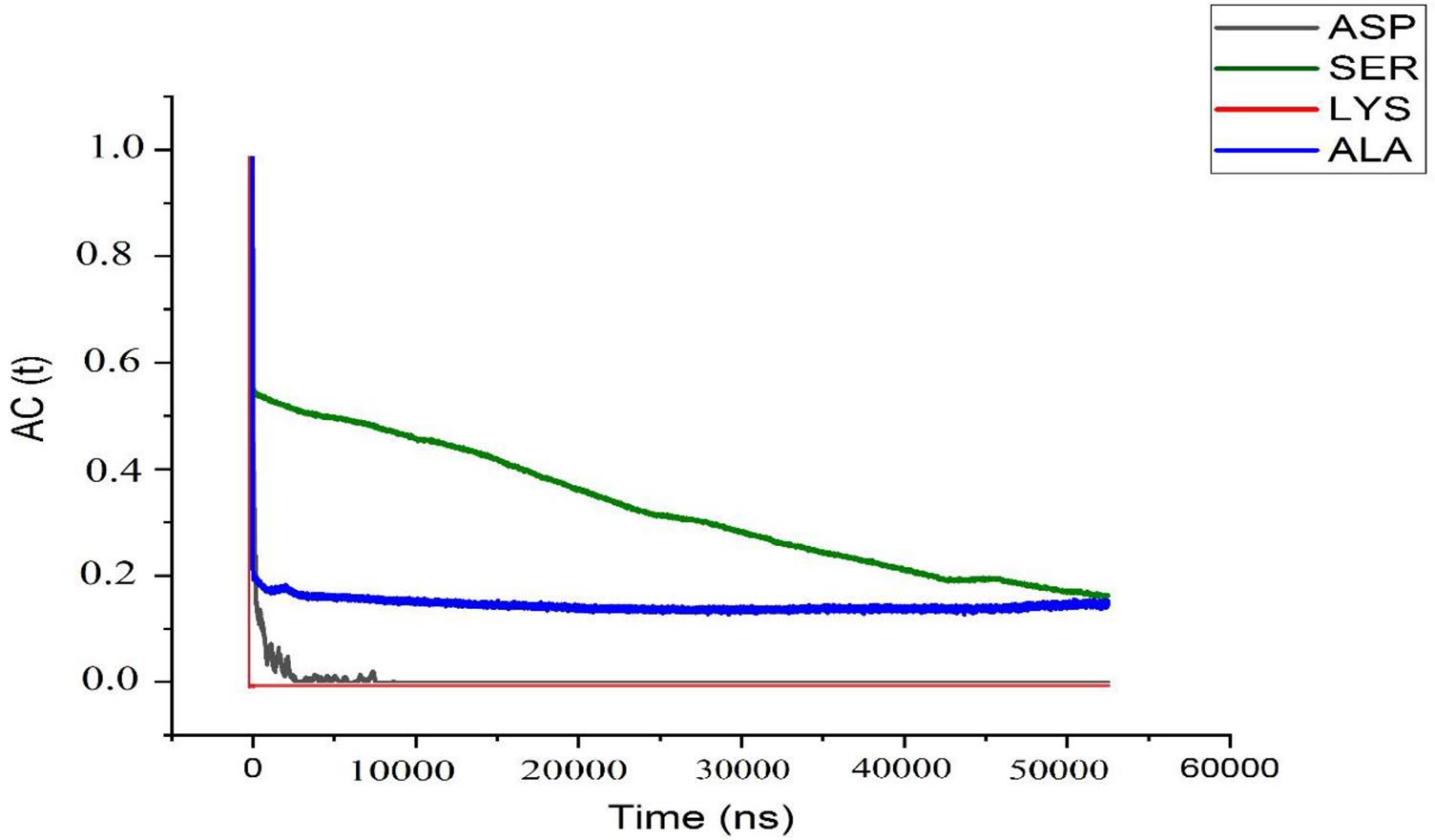
Hydrogen bond autocorrelation function between the amino acids and the carrier surface.

These results can be further explored through the calculation of the hydrogen bond autocorrelation function (HB-ACF). Through these calculations, a glimpse of hydrogen bond lifetime can be achieved. Results of HB-ACF calculations are provided in Fig. 7. In this figure, the lifetime of HB in the LYS-BNNS complex is shown to be zero, as there formed no HB in these complexes. In the same line, a very limited lifetime is observed for HBs in ASP-BNNS complexes, where the interactions between those components are repulsive, and the formation of HBs are rare. However, for the ALA-BNNS complex, there is the possibility to retain 20% of HBs up to 50 ns, and this finding complies with number of HBs in Fig. 5, where at least one HB could be seen through much of the simulation time. Finally, the SER-BNNS complex reveals a gradual decrease in the lifetime of HBs, a pattern that could also be seen in the number of pair interactions in the above-mentioned complex. Accordingly, the gradual reduce might be the direct consequence of the decrement in the number of possible interactions between the polar side group of the amino acid and the substrate.

**Figure 8:**
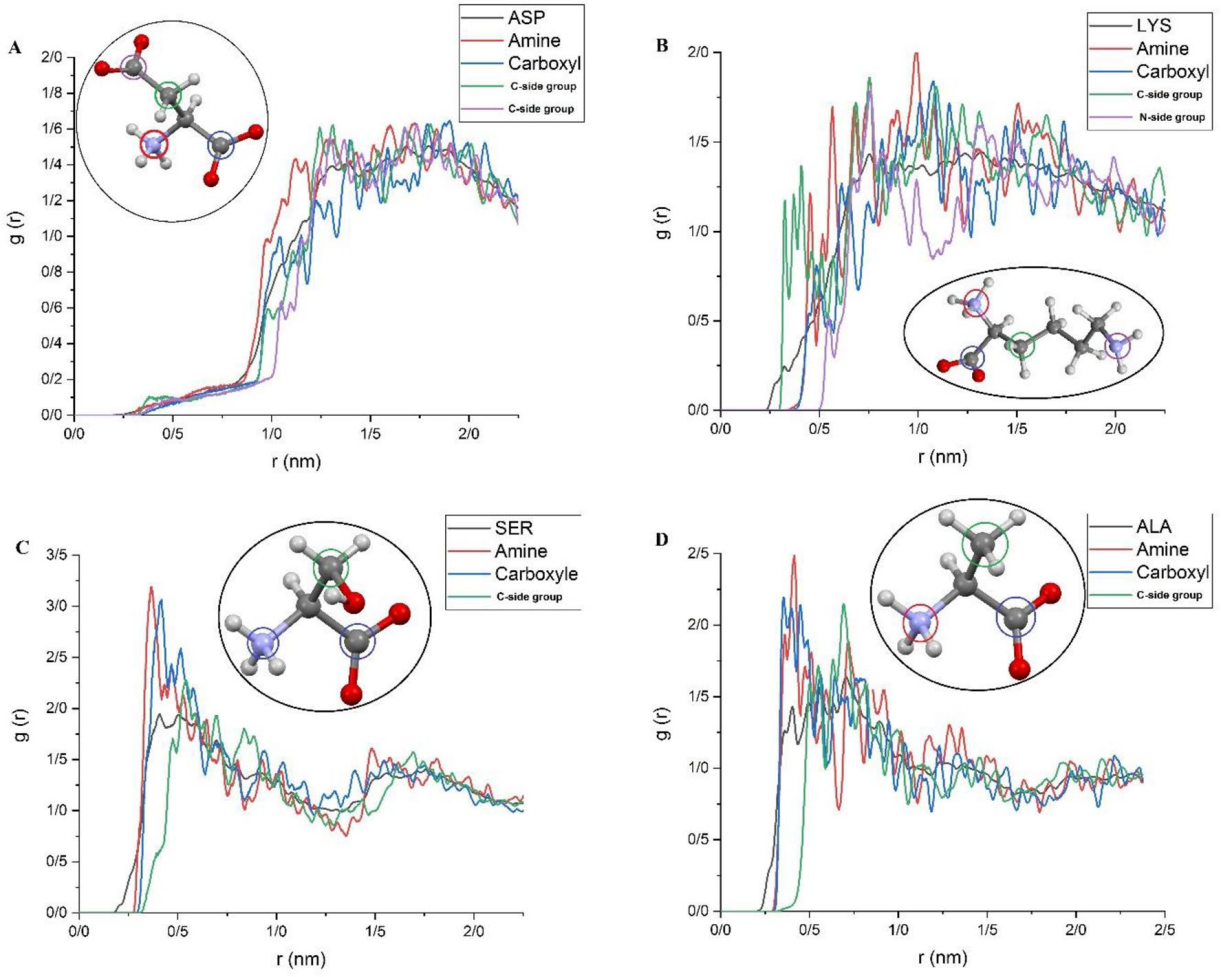
The atomic radial distribution function for any central atom in each active site in amino acids with respect to the carrier surface.

Each studied amino acid is composed of a backbone (amine and carboxyl groups) and a side group chain. To investigate the exact contribution of each active site of the amino acids in the adsorption of these molecules on the substrate, atomic RDFs for any central atom in each active site are calculated and presented in Fig. 8. Close inspection of this figure reveals that the height of aRDF for amine and carboxyl groups increases in closer distance to BNNS and locates at higher positions compared to RDF of the molecule of interest. This finding indicates that amine and carboxyl groups play the role of adsorption agents in the structure of amino acids. Nevertheless, for ASP molecules, the repulsive interaction between the negative charge point on the BNNS surface and the negatively charged side chain of the amino acid leads to its repulsion from the carrier. In fact, COO^−^ functional group causes the aRDF peak appears at further away distances compared to the RDF pattern of the ASP molecule and may contribute to the repulsion of the amino acid from the BNNS surface. While for LYS molecules that show the highest interaction energies, the carbon atom from the CH_2_ tail of its side group shows the closest peak to the BNNS surface, claiming the highest affinity toward the substrate. Considering that there are four CH2 groups in the side chain of the LYS molecule, it can be concluded that their amplified affinity toward BNNS contributes to the formation of the highest coulombic interactions in the investigated complexes.

### Metadynamics simulations

**Figure 9:**
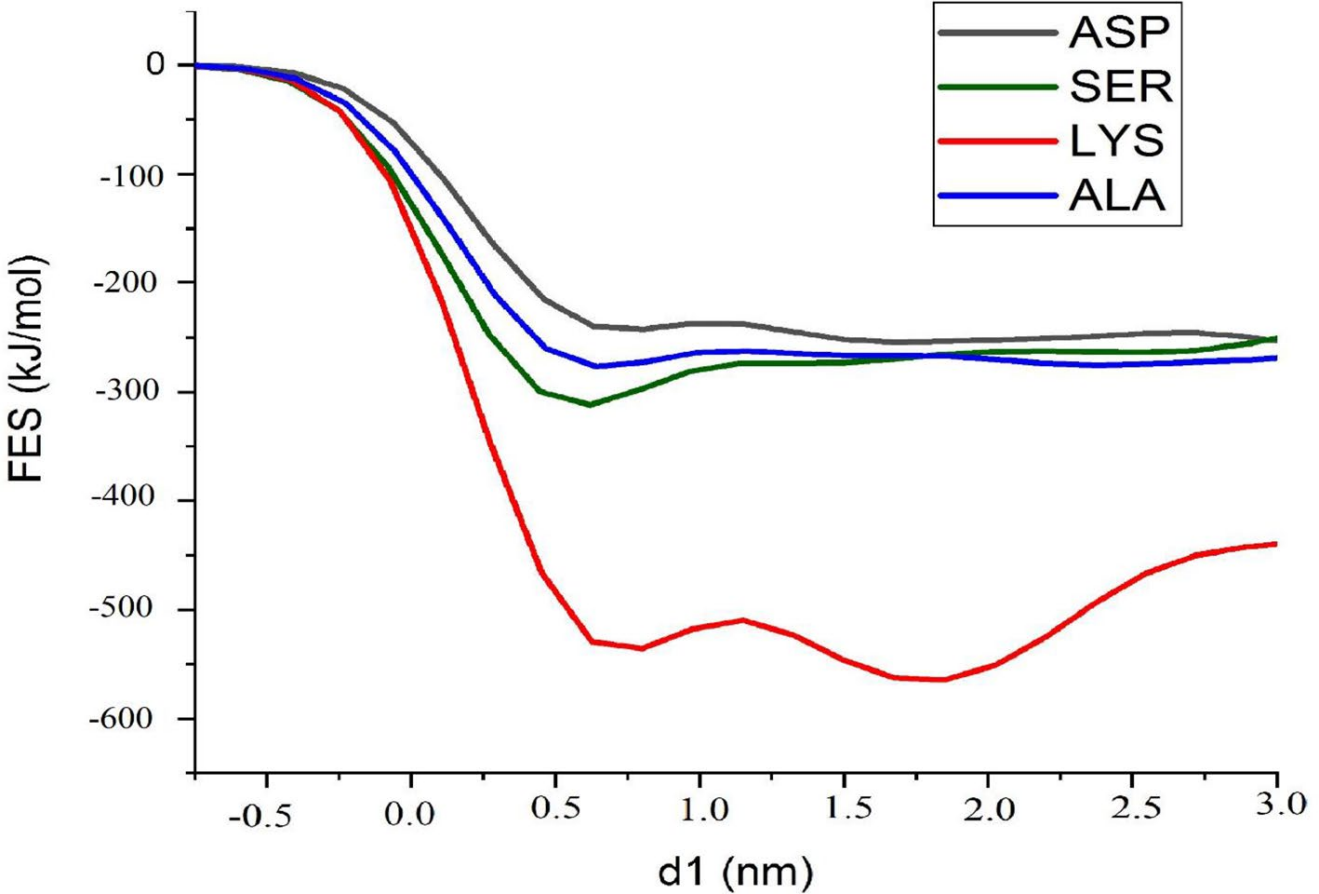
The free energy profile for the different adsorption of amino acids on the BNNS surface as a function of the center of mass of the molecules from the carrier.

Finally, Free energy surfaces are also investigated in order can be used as a decisive clue for confirming all the above-mentioned interactions and distribution patterns. Metadynamics simulations are performed for a system containing a single amino acid alongside a BNNS nanosheet. Resulted patterns are provided in Fig. 9 and show that the LYS-BNNS complex has the highest free energy at its stable state, confirming the interaction energies. There are no obvious global minimums for the ASP-BNNS complex in the metadynamics simulations, which remind ASP’s repulsive interactions toward the BNNS surface. The global minimum for SER and ALA are somewhere between ASP and LYS, but placed much closer to BNNS surface compared to LYS. It is unclear why ALA and SER have similar free energy landscapes and what causes such free energy landscapes for these two amino acids. To our understanding, this similarity may gain its origin from their smaller size. In summary, the smaller molecular size of the amino acids (ALA, ASP, and SER), in our setup, leads to the formation of a lower energy minimum, regardless of the origin of the deriving force for the adsorption, whether it is from the side chains or the backbone. As mentioned before, LYS has a slightly longer side chain and accordingly has a larger size than other amino acids (Fig. 1). This feature is reflected in its global minimum position, which is noticeably deeper and relatively farther away from the COM of the BNNS with respect to ALA and SER. This finding is also in agreement with the RDF calculations where the highest peaks for SER and ALA posed at around 0.5–0.7 nm away from the BNNS surface. While for the FES pattern, the global minimum (free energy) for these amino acids are situated at 0.62 nm (− 311/85 kJ/mol) and 0.64 nm (− 276/78 kJ/mol), respectively, Table 4. There is no obvious global minimum for ASP in the free energy landscape, while in the RDF pattern the highest peak is seen at more than 2 nm away from the BNNS surface. This phenomenon might be related to the free movements of ASP within the solution, as its interactions with BNNS are repulsive. For LYS that shows its RDF peak at around 1.0–1.4 nm, there is a local minimum at ~ 0.7 nm and a global minimum at 1.85 nm (− 564/40 kJ/mol).

**Table 4:** Deepest point (nm) and Free energy (kJ/mol) of the adsorption of single amino acid on BNNS.

Amino acids	Deepest point	Free energy
asp	1/50	− 252/04
ser	0/62	− 311/85
lys	1/85	− 564/40
ala	0/64	− 276/78

## Conclusion

A series of DFT calculations, MD simulations and another series of metadynamics simulations are carried out in this work to explore the possibility of amino acid adsorption on the boron nitride nanosheet. Selected amino acids have functional groups including polar, non-polar, negative, and positive charges, to gain deep insight into side groups of these compounds. Our results revealed the contribution of functional groups and backbone of the amino acids in the adsorption or repulsion features of the studied systems. In the DFT section, the obtained results for stable minimum energy configuration of the complexes concluded that the amino acids themselves orient parallel to the BNNS surface. Examinations related to the quantum chemistry reactivity parameters and adsorption energy values display the interaction of amino acids with BNNS nanosheet is energetically favorable in the solvent phase, and the type of adsorption process is physical. However, the physical nature of the adsorption process presents advantages in terms of easy elimination and reusability of nanosheet with no electronic and structural changes in the adsorbate and adsorbent. In addition, according to our DFT calculations, it appears that the structural parameters and the strength of the interaction of the configurations mainly depend on the nature of the side-chain of amino acids, and the polar amino acids have higher binding energy than nonpolar Ala.

The obtained interaction energies revealed that ASP molecules have repulsive interaction with BNNS nanosheet, and this finding is confirmed by the MSD and RDF analyzes. Moreover, three other amino acids show attraction forces toward BNNS. Among them, LYS molecules have the highest attractions, which are derived from their relatively higher coulombic interactions with BNNS However, the HB analysis confirmed that no HB (as coulombic interactions) formed between LYS and BNNS in the simulation, which is inconsistent with higher coulombic interactions.

RDF analysis suggested that the highest peak for distribution of ALA and SER laid at around 0.5 nm away from the BNNS surface, while the same peak for LYS is seen at 0.75 nm. These findings explain why there are limited chances for LYS to form HB interaction with the substrate. On the other hand, aRDF indicates that the amine and carboxyl groups in all the studied amino acids show relatively higher attraction toward the BNNS. Finally, it can be concluded that carboxyl and amine functional groups in amino acids tend to be adsorbed on the BNNS, and the overall behavior of the molecules toward the substrate is defined by its side chains. The LYS molecule showed a higher attraction toward BNNS because of its alkane tail in its side chain, and the ASP revealed the repulsion force originating from its COO– group. All the mentioned above suggestions are confirmed by free energy analyzes in which the LYS showed the highest adsorption free energy at a relatively farther distance concerning ALA-BNNS and SER-BNNS complexes. On the other hand, ASP showed no changes in its free energy curve as it approached the BNNS surface. The present work emphasizes that the BNNS nanosheet is an effective substrate to amino acids. The reliable conclusions drawn in this paper will encourage the experimentalists to explore and use these nanosheets as the amino acid carrier and to immobilize the amino acid.

## Supplementary Information

Supplementary Figure 1.
