# Author Correction: Interactions of boron nitride nanosheet with amino acids of differential polarity

**DOI:** 10.1038/s41598-025-16593-2

**Published:** 2025-08-29

**Authors:** Fatemeh Najafi, Farzaneh Farzad, Samaneh Pasban

**Affiliations:** Department of Chemistry, University of Birjand, Birjand, Iran

Correction to: *Scientific Reports* 10.1038/s41598-022-13738-5, published online 01 July 2022

The original version of this Article contained errors in the Computational methods section and in the References.

As a result, in the section ‘Computational methods’, in sub-section ‘Molecular dynamics simulations’:

"The CHARMM36 force-field model has been applied to all the studied systems^36^”

now reads:

“A detailed understanding of the force field parameters and atomic charges associated with the boron (B) and nitrogen (N) atoms focuses on two key parameters: sigma (σ), which indicates the distance where potential energy is zero and affects atomic proximity, and epsilon (ϵ), which measures the strength of attraction between atoms. Both parameters are essential for accurately simulating interactions in boron nitride. These parameters were extracted from studies conducted by Wu et al.^36^. Also, the parameters for amino acids utilized were obtained through the SwissParam web server (available https://www.swissparam.ch/)”

Additionally, in the References;

“Lee, J. et al. CHARMM-GUI input generator for NAMD, GROMACS, AMBER, OpenMM, and CHARMM/OpenMM simulations using the CHARMM36 additive force field. J. Chem. Theory Comput. 12, 405–413 (2016).”

now reads:

“Wu, Y., Wagner, L. K. & Aluru, N. R. Hexagonal boron nitride and water interaction parameters. J. Chem. Phys. 144, (2016).”

The original Article has been corrected.

