# Supplementary material for: Interactions of boron nitride nanosheet with amino acids of differential polarity
Source: Sci Rep. 2022 Jul 1;12:11156. doi: 10.1038/s41598-022-13738-5 (PMC9249799; doi:10.1038/s41598-022-13738-5)
Supplement: Supplementary file 1 — Supplementary Figure 1. [file 41598_2022_13738_MOESM1_ESM.docx]

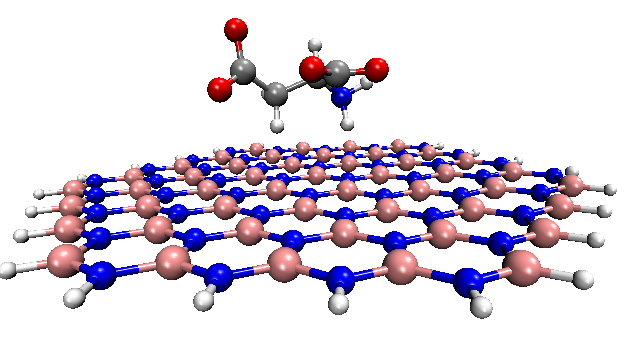

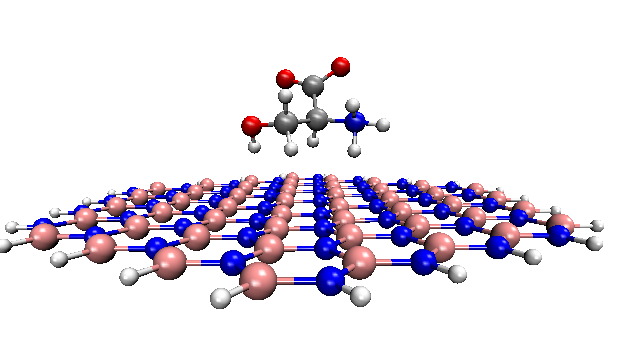

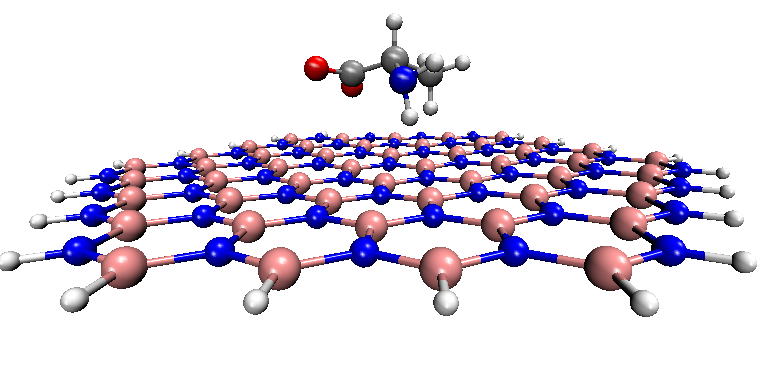

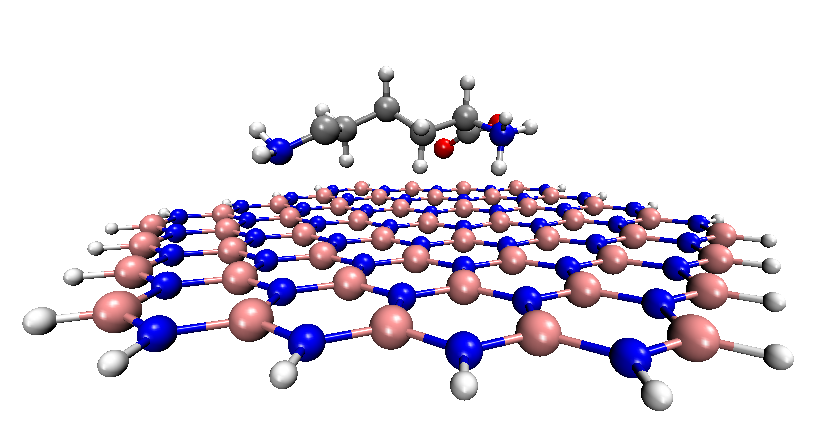


Ala-BNNS

Asp-BNNS

Ser-BNNS

Lys-BNNS

Figure S1. Optimized structures of the amino acid/BNNS complexes in the solvent phase at the M06-2X/6-31G** level, (atomic symbols: N (blue), O (red), C (gray), B (pink), and H (white)).
